# NCOA4 as a regulatory switch linking DNA damage to lipid peroxidation in radiation response

**DOI:** 10.3389/fcell.2026.1917345

**Published:** 2026-08-18

**Authors:** Pengju Yi, Ling Qiu, Hailing Han, Tiejun Wang, Yuqi Bai

**Affiliations:** The Second Hospital of Jilin University, Changchun, China

**Keywords:** ferritinophagy, ferroptosis, labile iron pool, lipid peroxidation, NCOA4, radiotherapy

## Abstract

Radiotherapy remains a cornerstone of cancer treatment; however, radioresistance and normal tissue toxicity continue to limit its therapeutic efficacy. Ferroptosis, an iron-dependent form of regulated cell death driven by lipid peroxidation, has emerged as an important determinant of radiation response and a promising target for radiosensitization. Among the regulatory mechanisms involved, NCOA4-mediated ferritinophagy has attracted increasing attention because it controls intracellular iron mobilization through selective ferritin degradation. Nevertheless, its role should be considered within the broader regulatory network governing radiation-induced ferroptosis. This review summarizes current evidence on the interplay between radiotherapy, iron metabolism, and ferroptosis, with emphasis on the regulatory contribution of NCOA4-mediated ferritinophagy. We discuss how radiation-induced DNA damage responses, oxidative stress, autophagy–lysosome pathways, and the tumor microenvironment collectively influence iron homeostasis and ferroptosis susceptibility. We further integrate recent advances in lipid remodeling, antioxidant defense systems, and the spatiotemporal dynamics of intracellular iron redistribution following irradiation. In addition, the interactions between ferroptosis and other radiation-induced cell death programs are highlighted to provide a comprehensive perspective on radiation-induced cellular responses. Finally, we critically evaluate the therapeutic potential and current limitations of targeting NCOA4-mediated ferritinophagy for radiosensitization. Rather than functioning as the sole determinant of radiation-induced ferroptosis, NCOA4 represents an important regulatory component within an integrated network of iron metabolism, oxidative stress, and lipid peroxidation. A better understanding of these interconnected mechanisms may facilitate the development of more effective and selective ferroptosis-based radiotherapeutic strategies.

## 1 Introduction

Cancer represents a major social, public health, and economic burden in the 21st century. Approximately one in five individuals develops cancer over their lifetime, and nearly one in nine men and one in twelve women ultimately die from this disease, rendering cancer one of the leading causes of premature mortality worldwide (Bray et al., 2024). Notably, cancer incidence has been increasingly reported in younger populations, underscoring an urgent demand for the development of more effective therapeutic strategies (Li et al., 2024a).

Radiotherapy remains a cornerstone of modern cancer treatment. Conventionally, its antitumor effects are understood to be primarily driven by ionizing radiation-induced DNA damage, oxygen radical-mediated lipid peroxidation, and the activation of canonical cell death pathways including apoptosis (Jiang et al., 2018). In recent years, accumulating evidence has demonstrated that ferroptosis, a regulated iron-dependent form of cell death, plays a critical role in tumor radiosensitivity (Li et al., 2020a). Since Dixon et al. formally defined ferroptosis in 2012 as a programmed cell death pathway distinct from apoptosis and necrosis, it has attracted growing attention as a promising therapeutic target for radiotherapy (Dixon et al., 2012).

During ferroptosis, iron, particularly mobilizable free iron, acts as a core catalyst for lipid peroxidation. Via the Fenton reaction and associated redox processes, free iron promotes the conversion of hydrogen peroxide into highly reactive hydroxyl radicals, which subsequently attack phospholipids enriched in unsaturated fatty acids (Mancias et al., 2014; Mancias et al., 2015). The extent of lipid peroxidation is tightly modulated by cellular antioxidant defense systems, including glutathione peroxidase 4 (GPX4), glutathione (GSH), and the system XC- cystine/glutamate antiporter (Tu et al., 2021; Stockwell and Jiang, 2022; Ye et al., 2020). Accordingly, the mobilization and regulation of intracellular iron stores serve as a fundamental determinant of the initiation and progression of ferroptosis (Lei et al., 2020; Zhou et al., 2022; Stockwell, 2022; Wang et al., 2023a; Cao and Dixon, 2016).

In the context of radiotherapy, emerging evidence indicates that ionizing radiation elevates lipid peroxidation while impairing intracellular antioxidant defenses, thereby increasing cellular susceptibility to ferroptosis (Ye et al., 2020; Tang et al., 2021; Doll et al., 2017). Although numerous studies have investigated the associations among radiation exposure, oxidative stress, and ferroptosis (Lei et al., 2022; Tang et al., 2021; Doll et al., 2017; Koppula et al., 2021), most of these studies remain descriptive. The detailed mechanistic cascade connecting radiotherapy-induced DNA damage to iron pool dysregulation, subcellular iron redistribution, lipid peroxidation, and eventual ferroptotic cell death has not been fully elucidated, leaving critical gaps in the understanding of the spatiotemporal regulation of ferroptosis during radiotherapy.

This review focuses on the mechanistic role of Nuclear Receptor Coactivator 4 (NCOA4)-mediated ferritinophagy as a regulatory switch in cellular responses to radiotherapy. We elaborate on how radiation-induced DNA damage triggers NCOA4-dependent ferritin degradation, which leads to the release of labile iron and its redistribution to specific subcellular compartments, particularly mitochondria, lysosomes, and plasma membrane domains. Such spatial reorganization of iron facilitates localized lipid peroxidation and ultimately initiates ferroptosis. By delineating this regulatory axis, this review aims to establish a conceptual framework and identify potential molecular targets for radiosensitization, treatment optimization, and normal tissue protection during radiotherapy.

## 2 Molecular basis of iron storage and ferritinophagy

Iron is essential for fundamental biological processes, including DNA synthesis, mitochondrial respiration, and cellular oxidative metabolism. However, excess iron can catalyze Fenton and related redox reactions, generating highly reactive hydroxyl radicals that damage nucleic acids, lipids, and proteins (Federico et al., 2022; Santana-Codina et al., 2022; Igarashi et al., 2022). Therefore, cellular iron homeostasis is maintained via the precise coordination of iron uptake, storage, utilization, and efflux.

Ferritin acts as the primary intracellular iron storage complex. It is a heteromeric nanocage composed of heavy (FTH1) and light (FTL) chains: FTL primarily facilitates ferritin assembly and structural stability, whereas FTH1 additionally exhibits ferroxidase activity that converts ferrous iron (Fe^2+^) to trivalent ferric ions (Fe^3+^) for stable storage within the ferritin cavity (Hoelzgen et al., 2024). Each ferritin complex can store up to approximately 4,500 iron atoms, thereby restricting the pro-oxidant activity of free iron involved in Fenton-type reactions(Irimia-Dominguez et al., 2020; Reutovich et al., 2022) Under metabolic stress or conditions of elevated iron demand, ferritin is selectively degraded via autophagy through a process termed ferritinophagy, which releases bioavailable iron for cellular utilization (Mancias et al., 2015; Kuno et al., 2022). The specificity of this process is mediated by a dedicated adaptor protein that links ferritin to the autophagic degradation machinery.

Ferroptosis is regulated not by a single linear pathway but by an interconnected regulatory network centered on iron metabolism, lipid remodeling, and antioxidant defense (Wang et al., 2023a; Sun et al., 2023; Wang et al., 2023c; Wang et al., 2022b). Within the iron metabolism module, transferrin receptor 1 (TfR1) mediates cellular iron uptake, ferritin sequesters excess intracellular iron, ferroportin (FPN/SLC40A1) facilitates iron export, and iron regulatory protein 1 (IRP1) coordinates iron homeostasis by regulating the translation of iron-responsive genes (Wang et al., 2023a; Sun et al., 2023).

At the lipid metabolism level, cellular susceptibility to ferroptosis is largely determined by the composition and oxidative sensitivity of membrane phospholipids. Acyl-CoA synthetase long-chain family member 4 (ACSL4) and lysophosphatidylcholine acyltransferase 3 (LPCAT3) facilitate the incorporation of polyunsaturated fatty acids (PUFAs) into membrane phospholipids. Notably, arachidonate lipoxygenase 15 (ALOX15) catalyzes phospholipid peroxidation, thereby driving membrane damage during ferroptosis (Sun et al., 2023; Wang et al., 2023c; Wang et al., 2022b).

Counteracting these pro-ferroptotic events, multiple antioxidant defense systems function in parallel to detoxify lipid peroxides. The canonical System Xc^−^-GSH-GPX4 axis eliminates phospholipid hydroperoxides through glutathione-dependent reduction, while the FSP1-CoQ10 pathway, DHODH-CoQH_2_ pathway, and GCH1-BH_4_ pathway constitute GPX4-independent ferroptosis defense systems that localize to distinct subcellular compartments (Sun et al., 2023; Wang et al., 2023c; Wang et al., 2022b). Collectively, these pathways establish multi-layered protection against excessive lipid peroxidation.

These metabolic modules do not function independently. Instead, intracellular iron availability critically modulates lipid peroxide propagation, while antioxidant systems determine whether oxidative damage accumulates beyond the threshold required for the execution of ferroptosis.

Accordingly, radiation-induced ferroptosis should be considered a systems-level response driven by the convergence of DNA damage signaling, iron mobilization, lipid remodeling, redox imbalance, as well as stress adaptation. It should not be recognized as a consequence of a single molecular event. Within the above-mentioned integrated regulatory network, NCOA4-mediated ferritinophagy serves as an upstream iron-amplifying mechanism instead of an independent ferroptotic pathway. By mobilizing ferritin-bound iron, NCOA4 can expand the labile iron pool, enhance Fenton chemistry, and provide catalytic iron for lipid peroxide generation. Thus, the threshold for ferroptosis can be reduced when lipid remodeling pathways are triggered, or when antioxidant defenses are impaired (Wang et al., 2023a; Sun et al., 2023; Wang et al., 2023c; Wang et al., 2022b; Chen et al., 2023b). Growing evidence further demonstrates that ionizing radiation induces ferroptosis via the coordinated regulation of iron metabolism, ROS production, and lipid peroxidation, wherein NCOA4-mediated ferritinophagy acts as a critical modulator of radiation-induced iron mobilization and ferroptotic sensitivity (Wang et al., 2023a; Chen et al., 2023b).

NCOA4 functions as a selective cargo receptor that mediates ferritinophagy by binding to cytosolic ferritin and targeting it to lysosomes for degradation, enabling the controlled release of ferritin-stored iron into the labile iron pool (Mancias et al., 2014; Mancias et al., 2015; Tu et al., 2021). NCOA4 is a 614-amino-acid protein (∼70 kDa) that was initially identified as an androgen receptor co-activator due to its capacity to enhance androgen-dependent transcription (Yeh and Chang, 1996; Heinlein et al., 1999).

Structurally, NCOA4 contains distinct N- and C-terminal domains, and its conserved C-terminal region directly interacts with FTH1, which is an indispensable step for ferritinophagy and ferroptosis induction (Mancias et al., 2014; Mancias et al., 2015; Kuno et al., 2022; Ohshima et al., 2022; Zhang et al., 2021a). Through this interaction, NCOA4 recruits ferritin to autophagosomes either directly or through adaptor proteins associated with ATG8 family members. Accordingly, ferritin degradation and the release of ferrous iron into the intracellular labile iron pool can be facilitated.

Under physiological conditions, NCOA4 levels can be regulated by an iron-dependent feedback mechanism. When intracellular iron is abundant, the E3 ubiquitin ligase HERC2 mediates NCOA4 ubiquitination and proteasomal degradation. Thus, ferritin turnover can be restricted. In contrast, iron deficiency or cellular stress inhibits HERC2 activity, stabilizes NCOA4 protein levels, and enhances ferritinophagy to mobilize iron and maintain cellular iron homeostasis (Mancias et al., 2015; Anandhan et al., 2023; Zhao et al., 2024a). Collectively, the NCOA4-ferritin-HERC2 axis acts as a dynamic rheostat that modulates intracellular iron availability.

Recent studies have validated that NCOA4-mediated ferritinophagy in erythroid cells is essential for hemoglobin synthesis by continuously supplying iron to mitochondria (Ryu et al., 2017; Santana-Codina et al., 2019; Nai et al., 2021; Aronova et al., 2021). Tumor cells exhibit elevated metabolic demand and persistent stress, and increased NCOA4 expression in these cells is strongly correlated with enhanced ferroptosis sensitivity. NCOA4 silencing impairs ferritin degradation. Consequently, iron sequestration and reduced susceptibility to iron-dependent oxidative damage can be triggered. Conversely, NCOA4 overexpression can accelerate ferritin turnover and increase vulnerability to iron-driven lipid peroxidation (Gryzik et al., 2021; Fang et al., 2021; Wu et al., 2023a).

NCOA4-mediated ferritinophagy exhibits tight association with redox control and various metabolic signaling pathways. Multiple accumulating studies have shown that the tumor suppressor p53 can repress transcription of the cystine/glutamate antiporter SLC7A11, while stabilizing NCOA4 protein under oxidative stress conditions. This biological process coordinates GSH depletion and cellular iron release, ultimately promoting the initiation of ferroptosis (Wu et al., 2023a; Jiang et al., 2015; Li et al., 2024b; Lei et al., 2021). Moreover, existing research demonstrates that HIF-1α can upregulate NCOA4 expression under ischemic status or chemical stabilization of hypoxia-inducible factor signaling, which helps sustain intracellular iron flux during hypoxic stress (Li et al., 2020b; Tian et al., 2024; Fuhrmann et al., 2020; Yuan et al., 2023). The combination of these signaling pathways makes NCOA4 a core regulatory factor that links oxygen availability, redox balance and iron metabolism to ferroptotic susceptibility.

Besides transcriptional level regulation, post-translational modification of NCOA4 serves as a key mechanism to modulate its function in ferroptosis. In response to DNA damage or metabolic stress, multiple kinases including ATM and AMPK are capable of phosphorylating NCOA4. This modification promotes NCOA4 localization in the cytoplasm, and further accelerates ferritin degradation as well as intracellular iron mobilization (Wu et al., 2023a; Ou et al., 2024; Du et al., 2024; Liu et al., 2024). On the contrary, oxidative damage occurring on lysosomal membranes will interrupt normal ferritinophagy progression, leading to ferritin accumulation inside cells and eventual development of ferroptosis resistance (Wei et al., 2022; Qi et al., 2024; Tsukida et al., 2022).

In addition, NCOA4-dependent ferritinophagy acts in close coordination with lipid metabolism regulation. By accelerating ferritin degradation, NCOA4 releases ferrous iron into the cellular labile iron pool, which provides sufficient catalytic iron for the occurrence of enzymatic lipid peroxidation (Mancias et al., 2015; Gryzik et al., 2021; Fang et al., 2021; Lai et al., 2025; Wenzel et al., 2017; Shah et al., 2018). In this regulatory process, ACSL4 activates long-chain PUFAs and incorporates these fatty acids into membrane phospholipids, forming PUFA-PE species that are highly susceptible to peroxidation. These lipid molecules can be further oxidized by ALOX15/15-LOX to generate functional lipid signals that trigger ferroptosis. Such coordinated biological processes connect iron mobilization with lipid peroxidation, forming a feed-forward loop that significantly amplifies ferroptotic cell death. Subcellular imaging and proximity-labeling experimental data further prove that NCOA4 accumulates at ER-lysosome contact sites. This spatial distribution enables effective coordination between intracellular iron flux and phospholipid oxidation, ensuring efficient ferroptosis execution (Mancias et al., 2015; Wen et al., 2024; Özkan et al., 2021; Wenzel et al., 2022).

Although NCOA4-mediated ferritinophagy has recently attracted attention in the context of radiotherapy-induced ferroptosis, its biological function was initially established as a general mechanism responsible for intracellular iron recycling under diverse cellular stress conditions. Increasing evidence indicates that NCOA4 functions as a conserved stress-responsive regulator linking ferritin degradation, iron availability, and ferroptotic susceptibility beyond radiation-associated settings (Mancias et al., 2015; Anandhan et al., 2023; Petriaggi et al., 2025).

Under nutrient starvation and metabolic stress conditions, NCOA4-mediated ferritinophagy contributes to iron mobilization by selectively delivering ferritin to lysosomes for degradation. This process provides a bioavailable iron source that supports cellular metabolic adaptation and maintains iron homeostasis during periods of limited extracellular iron availability (Anandhan et al., 2023; Petriaggi et al., 2025). However, persistent activation of ferritinophagy may increase the LIP, enhance Fenton chemistry, and promote lipid peroxidation when antioxidant capacity is insufficient, thereby sensitizing cells to ferroptotic death (Gryzik et al., 2021; Petriaggi et al., 2025).

Hypoxia represents another important physiological stress condition regulating NCOA4 activity. Under hypoxic environments, stabilization of hypoxia-inducible factor signaling can modulate iron metabolism-related genes and influence NCOA4-dependent ferritin turnover, thereby maintaining intracellular iron circulation and mitochondrial metabolic function (Li et al., 2020b; Petriaggi et al., 2025). However, when hypoxia is accompanied by oxidative stress or impaired antioxidant defense, excessive iron mobilization may shift the cellular balance toward ferroptotic vulnerability.

In addition to metabolic and oxygen-related stress, environmental and pharmacological stresses can also interact with the NCOA4-ferritinophagy axis. Heavy-metal exposure and drug-induced oxidative stress disturb cellular redox homeostasis and may increase ferroptosis sensitivity by altering iron metabolism, mitochondrial function, and lipid peroxide accumulation (Shah et al., 2018; Petriaggi et al., 2025). In particular, pharmacological inhibition of ferroptosis defense systems, such as GPX4 suppression, can cooperate with NCOA4-mediated iron release to accelerate iron-dependent lipid peroxidation (Gryzik et al., 2021; Wen et al., 2024).

Collectively, these findings demonstrate that NCOA4-mediated ferritinophagy represents a broad stress-adaptive iron regulation program rather than a radiation-specific mechanism. Radiation-induced activation of NCOA4 should therefore be interpreted as one context-dependent manifestation of a conserved cellular response that integrates iron recycling, oxidative stress, and ferroptotic regulation. Understanding the common regulatory principles shared between radiation and non-radiation stress models will be essential for accurately defining the therapeutic potential and limitations of targeting NCOA4 in cancer treatment (Wang et al., 2023a; Petriaggi et al., 2025).

**FIGURE 1:**
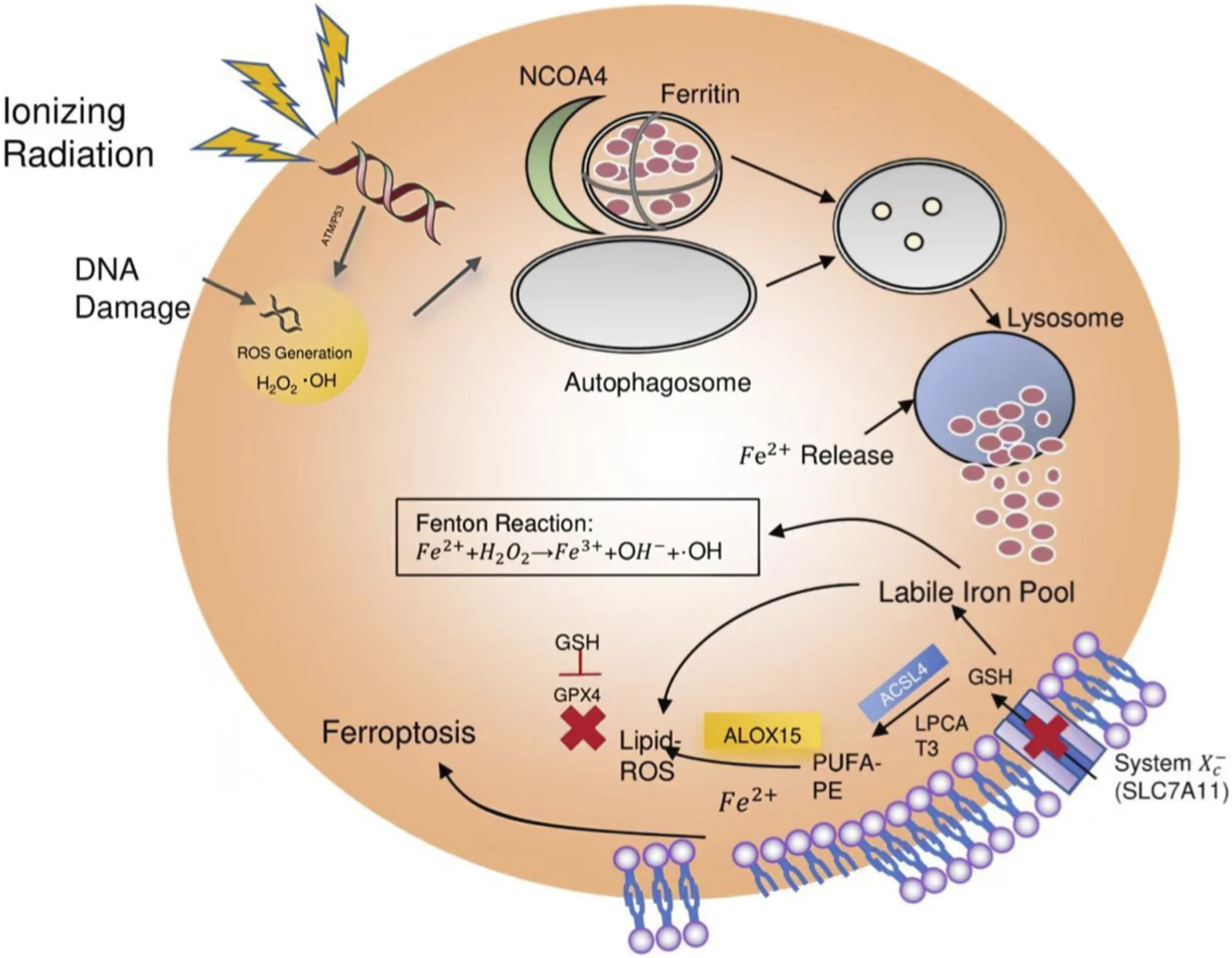
Mechanistic overview of radiation-induced ferroptosis highlighting the regulatory role of NCOA4-mediated ferritinophagy. Ionizing radiation induces multiple cellular stress responses, including reactive oxygen species (ROS) generation and DNA damage. DNA damage signaling, including ATM–p53 pathway activation, may contribute to NCOA4 stabilization under specific conditions. Activated NCOA4 facilitates ferritin trafficking to lysosomes and ferritin degradation, resulting in increased availability of labile iron. Released Fe^2+^ enhances iron-dependent oxidative reactions and cooperates with lipid remodeling pathways, including ACSL4-, LPCAT3-, and ALOX15-mediated phospholipid peroxidation. Meanwhile, radiation-associated impairment of antioxidant systems, such as the System Xc^−^–GSH–GPX4 axis, increases cellular susceptibility to lipid peroxide accumulation. Collectively, these interconnected processes contribute to radiation-induced ferroptosis, with NCOA4-mediated ferritinophagy representing one important regulatory mechanism within the broader ferroptosis network.

Taken together, currently available evidence suggests that NCOA4-mediated ferritinophagy acts as a context-dependent regulatory node. This node connects DNA damage response, iron metabolism, phospholipid remodeling and cellular antioxidant defense systems. Instead of independently driving ferroptosis progression, NCOA4 collaborates with a wide range of ferroptosis-related pathways to modulate cell susceptibility to ferroptosis under radiation-induced stress. However, the regulatory role of NCOA4 is highly context-specific. Its function varies depending on tumor type, radiation treatment modality, cellular metabolic state, and the activity of other parallel ferroptosis regulatory pathways. These findings provide a solid conceptual basis for understanding tumor radiosensitization and screening effective targets for clinical combination therapy (Figure 1).

## 3 Upstream signaling mechanisms regulating radiation-induced ferritinophagy

Ionizing radiation can induce multiple cytotoxic cellular responses, such as DNA double-strand breaks (DSBs), protein oxidation and mitochondrial dysfunction. DNA damage is traditionally recognized as the core factor determining radiotherapy efficacy. Nevertheless, a growing number of studies reveal that radiation-triggered oxidative stress and ferroptotic cell death also play essential roles in regulating tumor therapeutic responses (Wang et al., 2023b; Lang et al., 2019). In this research context, the crosstalk between radiation-induced reactive oxygen species (ROS) and NCOA4-mediated ferritinophagy is regarded as a vital but incompletely explored regulator of ferroptosis susceptibility.

During radiotherapy, ionizing radiation triggers the radiolysis of intercellular water and produces ROS including hydroxyl radicals and hydrogen peroxide. These reactive species not only drive lipid oxidation but also facilitate redox cycling between intracellular ferrous and ferric iron (Lang et al., 2019; Svoboda et al., 2020). The resultant oxidative stress disrupts cellular iron homeostasis by inducing lysosomal membrane permeabilization, which releases stored iron into the cytosol and facilitates ferritin degradation (Fernández et al., 2022; Reinheckel and Tholen, 2022). Notably, experimental studies have confirmed that ionizing radiation expands the intracellular labile iron pool within minutes, prior to the detection of lipid peroxidation. This temporal characteristic indicates that radiation-triggered iron mobilization acts as an initiating event, instead of a secondary outcome, during ferroptotic cell death (Delanoy et al., 2024).

NCOA4 acts as a core mediator of radiation-induced iron mobilization. Ionizing radiation elevates NCOA4 expression at both transcriptional and translational levels, which promotes ferritin delivery to autophagosomes and accelerates ferritinophagy progression. The subsequent release of ferrous iron sustains Fenton reactions, causing excessive hydroxyl radical production, aggravated lipid peroxidation, and gradual loss of cell membrane integrity (Mancias et al., 2015; Zhou et al., 2022; Wu et al., 2023a). In line with this regulatory mechanism, genetic ablation of NCOA4 or pharmacological blockade of autophagy (e.g., chloroquine or 3-MA) substantially inhibits radiation-induced iron accumulation and induces prominent radioresistance in multiple cancer models (Zhou et al., 2022; Gryzik et al., 2021; Du et al., 2024; Li et al., 2023).

Radiation-induced DNA damage rapidly activates ATM kinase, which triggers signaling cascades to stabilize p53 and coordinate DNA repair with metabolic adaptation (Zhao et al., 2024b). Beyond its canonical role in genome maintenance, p53 serves as a critical regulator of ferroptosis, with highly context-dependent biological effects. Under specific conditions, p53 promotes ferroptosis by repressing the transcription of the cystine/glutamate antiporter SLC7A11. This suppression limits cystine uptake, depletes intracellular glutathione (GSH), and impairs GPX4-dependent antioxidant defenses (Dixon et al., 2012; Jiang et al., 2015; Yang et al., 2014). p53 can also increase cellular ferroptotic susceptibility via additional metabolic and oxidative stress-associated pathways. Emerging data further suggest that ATM signaling modulates NCOA4-mediated ferritinophagy through the coordination of DNA damage responses, oxidative stress, and autophagy-related processes. Nevertheless, whether ATM directly targets and regulates NCOA4 across diverse tumor types and radiation conditions remains incompletely clarified (Zhou et al., 2022; Wu et al., 2023a; Zhao et al., 2024b; Zeng et al., 2024). Collectively, these results demonstrate a functional correlation among ATM signaling, p53 activation, and NCOA4-mediated ferritinophagy, rather than a fixed and universal linear signaling cascade.

Notably, the role of p53 in ferroptosis is not consistently pro-ferroptotic. Depending on cell type, stress magnitude, and downstream transcriptional programs, p53 may promote ferroptosis by modulating target genes such as SLC7A11 and SAT1. Conversely, it can also suppress ferroptotic cell death by activating protective pathways, including CDKN1A (p21)-mediated cell-cycle arrest and metabolic adaptation. As such, the regulatory effect of the ATM-p53-NCOA4 axis on radiation-induced ferroptosis is highly context-dependent and varies among different tumor models and irradiation conditions. Current evidence validates that NCOA4-mediated ferritinophagy is a key regulatory component of this signaling network, while further investigations are required to elucidate its hierarchical relationship with ATM and p53 signaling pathways.

Lysosomes serve as the central platform for ferritinophagy, and ionizing radiation profoundly regulates lysosomal dynamics. Radiation activates transcription factor EB (TFEB), a master regulator of lysosomal biogenesis and autophagy-related gene expression. Thus, lysosomal capacity can be expanded (Chen et al., 2023a). Under moderate levels of radiation-induced ROS, TFEB nuclear translocation can be promoted, NCOA4 expression can be upregulated, and ferritin degradation with subsequent iron release can be accelerated (Curnock et al., 2023; Chen et al., 2023a). In contrast, excessive oxidative stress damages lysosomal membranes, disrupts autophagic flux, and paradoxically triggers ferritin accumulation, ultimately reducing cellular susceptibility to ferroptosis (Zhang et al., 2023a). These findings demonstrate that radiation-induced ferritinophagy is controlled by a narrow redox window: insufficient ROS promotes iron sequestration and cell survival, whereas excessive ROS impairs lysosomal function and restricts iron mobilization. Lysosomal activation and NCOA4-dependent iron release can effectively drive ferroptotic cell death only in an intermediate oxidative range.

Mitochondria-lysosome contact sites constitute an additional regulatory node in radiation-induced ferritinophagy. High-resolution imaging and proximity-labeling proteomic analyses have verified that ionizing radiation induces NCOA4 redistribution to ER-lysosomal and mitochondrial-lysosomal interfaces, facilitating the targeted transfer of ferritin-derived ferrous iron to mitochondria (Mancias et al., 2014; Zhang et al., 2023a; Fujimaki et al., 2019; Ito et al., 2021). Mitochondrial iron loading further enhances ROS production via the tricarboxylic acid cycle and the electron transport chain. The above-mentioned spatially coordinated iron trafficking forms a feed-forward oxidative amplification loop. On that basis, radiation-activated NCOA4 can promote mitochondrial iron accumulation and increase vulnerability to membrane lipid peroxidation, especially at the nuclear envelope.

The hypoxic tumor microenvironment (TME) further modulates cellular responses to radiation-induced ferroptosis. Under hypoxic conditions combined with radiation-associated oxidative stress, hypoxia-inducible factor-1α (HIF-1α) remains stabilized and transcriptionally upregulates NCOA4 along with key iron-handling proteins, including divalent metal transporter 1 (DMT1) and transferrin receptor (TFRC) (Wang et al., 2010; Su et al., 2022). This adaptive program maintains intracellular iron flux and supports cellular metabolic adaptation. Consistently, studies based on glioblastoma and hepatocellular carcinoma models confirm that HIF-1α stabilization synergizes with ionizing radiation to enhance ferritin turnover and lipid peroxidation, thereby increasing ferroptotic susceptibility (Ye et al., 2020; Wang et al., 2010; Su et al., 2022).

**FIGURE 2:**
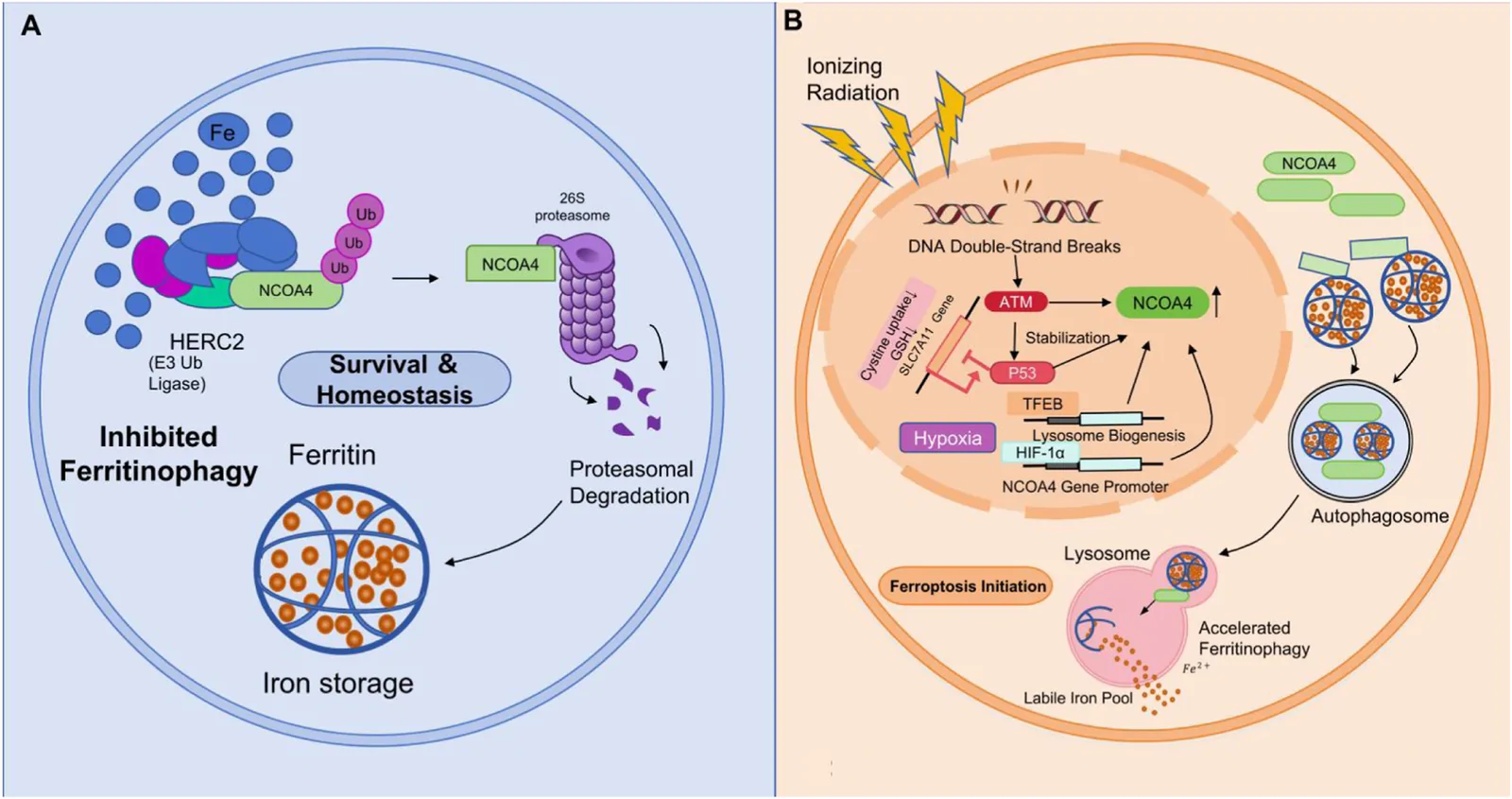
Context-dependent regulation of NCOA4-mediated ferritinophagy during iron homeostasis and radiation stress. Under physiological iron-replete conditions, HERC2-mediated ubiquitination promotes NCOA4 degradation, thereby limiting ferritinophagy and maintaining intracellular iron storage. Following irradiation, multiple stress-responsive pathways may regulate NCOA4 activity. ATM–p53 signaling has been implicated in NCOA4 stabilization, whereas oxidative stress, TFEB-associated lysosomal responses, and hypoxia-related pathways may further influence ferritinophagy activity in a context-dependent manner. Enhanced NCOA4-mediated ferritin degradation increases labile iron availability and may promote ferroptotic vulnerability. These regulatory interactions highlight NCOA4 as an important modulator of iron metabolism rather than an exclusive determinant of radiation-induced ferroptosis. **(A)** Physiological/Iron-Replete state. **(B)** Radiotherapy-Induced Stress Ferroptotic Switch.

Furthermore, radiation-induced depletion of NADPH and GSH significantly impairs the cellular ability to neutralize lipid peroxides generated via ferrous iron-dependent oxidative reactions (Ye et al., 2020; Shimura et al., 2022; Gansemer et al., 2020). The combined effects of excessive ROS accumulation, radiation-driven iron overload, and impaired antioxidant defenses construct a metabolic milieu conducive to ferroptosis initiation. Within this mechanistic framework, NCOA4-dependent iron mobilization acts as a core regulatory axis that bridges radiation-induced metabolic reprogramming and ferroptotic cell death (Figure 2).

## 4 Spatiotemporal dynamics of intracellular iron redistribution following ferritinophagy

Following radiation-induced activation of NCOA4-mediated ferritinophagy, intracellular iron distribution undergoes rapid, highly dynamic reorganization. The reorganization is temporally regulated and spatially compartmentalized. Rather than a static state of iron overload, accumulating evidence indicates that the timing, localization, and intracellular trafficking of labile iron are critical determinants of susceptibility to iron-dependent cell death (Mancias et al., 2015; Wu et al., 2023a; Fujimaki et al., 2019; Ito et al., 2021; Gao et al., 2016; Kagan et al., 2017; Chabane et al., 2026). As demonstrated by recent studies, endogenous glutamate modulates ferroptosis sensitivity via ADCY10-dependent inhibition of YAP signaling in lung adenocarcinoma, which further highlights the complexity of iron-regulated ferroptotic pathways (Zhang et al., 2021b; Wang et al., 2022a). The early phase post irradiation is characterized by acute oxidative stress and lysosomal destabilization, leading to rapid NCOA4-dependent release of ferritin-bound iron into the labile iron pool (Mancias et al., 2014; Zhou et al., 2022; Wu et al., 2023a; Reinheckel and Tholen, 2022; Wu et al., 2023b). This initial iron mobilization intensifies Fenton reactions at lysosomal-mitochondrial contact sites and generates spatially restricted lipid peroxidation foci, which subsequently propagate throughout the cytoplasm as progressive ferroptotic waves (Zhou et al., 2022; Fujimaki et al., 2019; Kagan et al., 2017; Co et al., 2024; Saimoto et al., 2025).

Temporally, ferritinophagy triggers a biphasic pattern of intracellular iron redistribution. An early transient surge of ferrous iron occurs within minutes to 1 hour after irradiation (Cosialls et al., 2023), followed by a delayed phase spanning approximately 4–12 h, during which iron preferentially accumulates in the mitochondria and perinuclear region (Zhou et al., 2022; Li et al., 2023; Yu et al., 2022). As indicated by the result of time-resolved imaging using FeRhoNox and FerroOrange probes, this delayed mitochondrial iron loading coincides with reduced GPX4 activity and elevated lipid ROS production (Yu et al., 2022). Mechanistically, the early iron wave is primarily driven by autophagosome-lysosome fusion and ferritin degradation. It is noteworthy that the late-phase iron accumulation reflects enhanced mitochondrial iron uptake mediated by mitochondrial ferritin two under oxidative stress and transient mitochondrial membrane potential disruption (Mancias et al., 2014; Mancias et al., 2015; Wang et al., 2021). As revealed by the above-mentioned findings, ferritinophagy not only increases intracellular iron bioavailability but also temporally orchestrates ferroptosis progression by synchronizing iron release kinetics with subcellular redox thresholds.

Spatially, ferritinophagy profoundly remodels the intracellular iron topology in irradiated cells (Zhou et al., 2022; Yang et al., 2023). Iron released from degraded ferritin is rapidly transported to mitochondria, where it facilitates iron-sulfur cluster repair but also exacerbates mitochondrial lipid peroxidation, particularly in the phosphatidylserine-rich inner mitochondrial membrane (Mancias et al., 2014; Kagan et al., 2023). Concurrently, radiation-induced lysosomal membrane permeabilization enables partial leakage of ferrous ions into the cytosol, which enhances localized lipid oxidation at perinuclear ER-mitochondrial contact sites (Saimoto et al., 2025; Ma et al., 2021). This iron redistribution process is further modulated by iron chaperone proteins that buffer excess cytosolic iron and direct iron trafficking toward subcellular compartments vulnerable to oxidative damage (Patel et al., 2019). X-ray fluorescence and fluorescence-based imaging studies confirm that iron and other metal ions exhibit polarized subcellular distributions that colocalize with oxidative hotspots (Bolitho et al., 2021). Consistently, irradiated tumor cells display coordinated iron redistribution and augmented lipid peroxidation, underscoring the tight coupling between iron trafficking and the spatial patterning of membrane oxidation (Ma et al., 2021).

Importantly, the spatial heterogeneity underlies the radiation-induced bystander effect. Iron-enriched regions generate iron-dependent ROS and lipid peroxidation products that diffuse into neighboring non-irradiated cells, thereby propagating ferroptotic signals (Ito et al., 2021; Co et al., 2024; Wang et al., 2024; Ariyoshi et al., 2019). NCOA4 silencing abolishes this wave-like spread of lipid peroxidation. Therefore, ferritinophagy can be highlighted as a temporal gatekeeper and a spatial amplifier of radiation-induced ferroptosis (Zhou et al., 2022; Gryzik et al., 2021; Co et al., 2024). Moreover, live-cell imaging of ferrous ions combined with redox-sensitive GFP reporters reveals a vectorial iron flux from lysosomes to mitochondria and subsequently to the ER, mirroring gradients of lipid ROS accumulation (Rizzoll et al., 2025; Pang et al., 2021). The above-mentioned directed transport is mediated by dynamic organelle contacts. On that basis, the ER and mitochondria coordinate iron exchange via VDAC1 and the mitochondrial ferritin complex. Accordingly, oxidative feedback loops can be reinforced (Zhou et al., 2022; Shiiba et al., 2025; Karmi et al., 2022).

**FIGURE 3:**
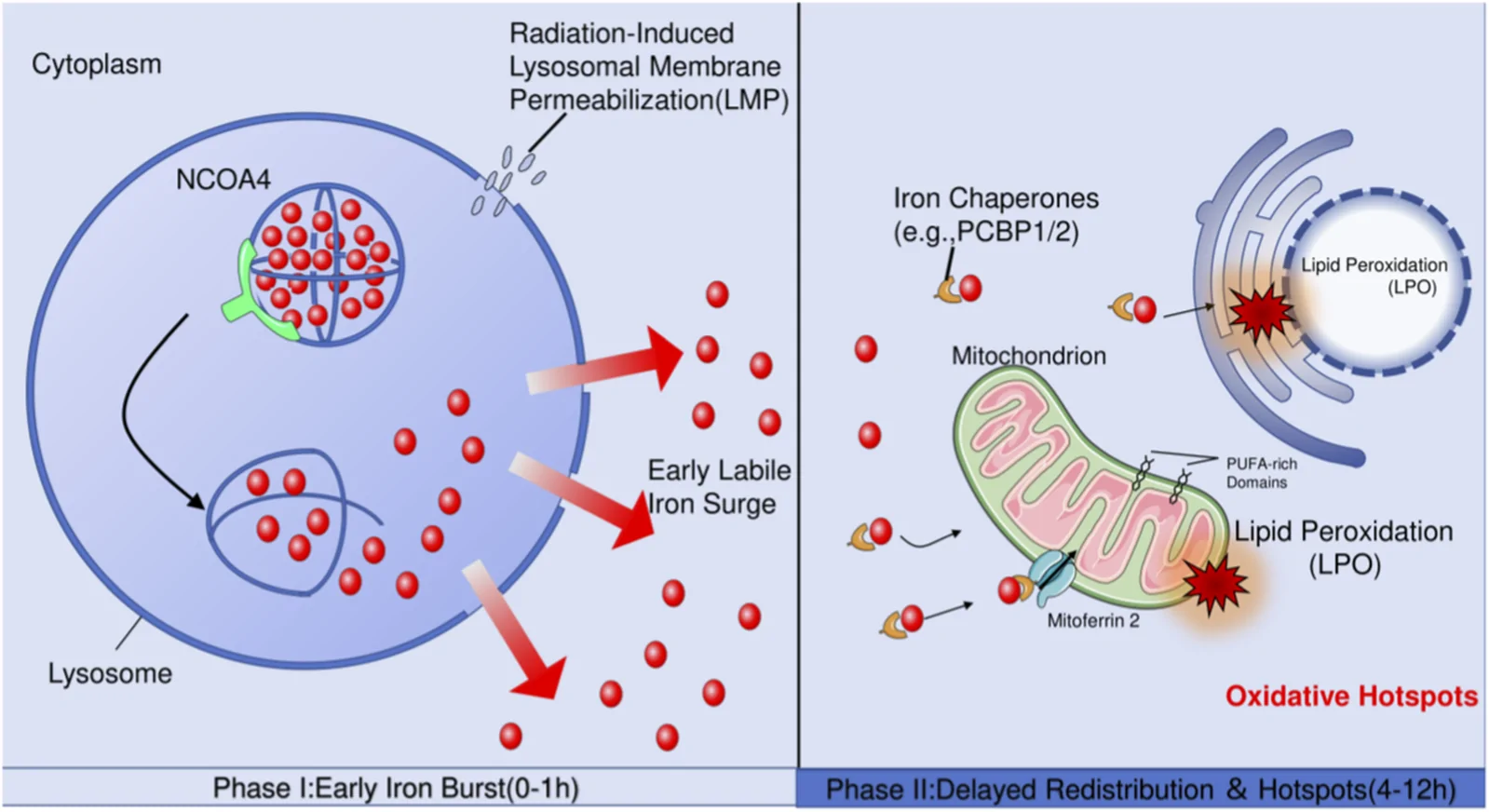
Proposed spatiotemporal dynamics of intracellular iron redistribution following radiation exposure. Radiation-induced ferritinophagy contributes to an early increase in intracellular labile iron availability through lysosomal ferritin degradation. Radiation-associated oxidative stress may also promote lysosomal membrane alterations, facilitating additional iron release. During subsequent stages, intracellular iron may undergo redistribution toward mitochondria and other subcellular compartments. Emerging evidence suggests that iron chaperones, mitochondrial iron transport systems, and organelle contact sites may participate in regulating localized iron trafficking. Accumulation of redox-active iron within lipid-rich membrane regions may promote localized lipid peroxidation and oxidative amplification. This model summarizes current hypotheses regarding dynamic iron remodeling after irradiation, while the precise mechanisms controlling organelle-specific iron trafficking require further investigation.

In brief, NCOA4-driven ferritinophagy converts iron from a passive redox component into a spatiotemporal signaling mediator of ferroptosis under radiation stress. This framework redefines ferroptosis not simply as a consequence of iron excess, but as the outcome of organized iron trafficking synchronized with lipid peroxidation kinetics and subcellular vulnerability. Elucidating these iron dynamics bridges radiation physics and cellular biochemistry and suggests that targeting time-dependent iron fluxes or compartment-specific iron transport pathways may enable spatially controlled ferroptosis and improve precision in radiotherapy (Figure 3).

## 5 Molecular mediators and effectors of ferritinophagy-driven ferroptosis

The spatiotemporally redistributed iron pool described above acts as a catalytic engine. Its lethal potential is realized only when coupled with a permissive lipid metabolic landscape and compromised antioxidant defenses, which are detailed in the following section.

The execution of ferroptosis following ferritinophagy activation is governed by a tightly integrated network of molecular effectors that couple iron metabolism with lipid peroxidation signaling. Once ferritin is degraded via NCOA4-dependent autophagy, the released ferrous iron serves as the principal catalyst for Fenton and Fenton-like reactions, initiating selective lipid peroxidation at phospholipid sites enriched in PUFAs (Dixon et al., 2012; Mancias et al., 2014; Mancias et al., 2015). However, the transition from iron release to complete ferroptotic cell death involves multiple intermediate regulatory layers, including the iron redox cycle, lipid metabolism remodeling, and the collapse of antioxidant defenses.

Following ferritin degradation, liberated ferrous ions are rapidly oxidized by hydrogen peroxide, generating highly reactive hydroxyl radicals that directly attack membrane phospholipids (Dixon et al., 2012; Mancias et al., 2014). As demonstrated by live-cell imaging using ferrous iron sensors and lipid peroxidation reporters, this redox activity is spatially heterogeneous, with maximal radical generation occurring near lipid bilayers and mitochondrial membranes that are intrinsically susceptible to oxidative damage (Anandhan et al., 2023; Ito et al., 2021; Kagan et al., 2017; Fellows et al., 2023). Lipoxygenases further amplify iron-driven oxidative signaling. In particular, ALOX15 utilizes ferrous iron as a cofactor to oxygenate PUFAs within phosphatidylserine, producing 15-hydroperoxy-eicosatetraenoyl-phosphatidylserine (15-HpETE-PE), a critical effector lipid of ferroptosis (Wenzel et al., 2017; Kagan et al., 2017; Ivanov et al., 2015). Ionizing radiation intensifies this cascade by upregulating ALOX15 expression and promoting its localization to oxidatively stressed mitochondria, thereby forming spatially confined pro-ferroptotic microdomains in irradiated tumor cells (Wenzel et al., 2017; Yamada et al., 2024).

Cells that are highly sensitive to ferroptosis exhibit a characteristic lipidomic profile enriched in long-chain PUFAs, including arachidonic and adrenic acids. ACSL4 catalyzes the activation of these fatty acids into their acyl-CoA derivatives, which are subsequently incorporated into membrane phospholipids by LPCAT3, generating a pool of readily oxidizable substrates (Lai et al., 2025; Reeves et al., 2021). Following irradiation, ACSL4 is transcriptionally upregulated via the p53-SLC7A11 axis and redistributes to ER-mitochondrial contact sites, facilitating the targeted enrichment of peroxidation-prone lipids in vulnerable membrane regions (Jiang et al., 2015; Ji et al., 2022). Functionally, the ACSL4-LPCAT3-ALOX pathway acts as a core biochemical conduit that converts ferritinophagy-derived iron flux into lipid peroxides to mediate ferroptotic damage. Consistently, genetic or pharmacological inhibition of ACSL4 markedly attenuates radiation-induced ferroptosis despite persistent NCOA4-mediated ferritin degradation, identifying ACSL4 as an essential lipid metabolic effector downstream of iron mobilization (Zhou et al., 2022; Lai et al., 2025; Ji et al., 2022).

Although iron initiates lipid peroxidation, the progression of this process to lethal levels relies critically on the failure of the GPX4-GSH antioxidant system, which reduces lipid hydroperoxides to non-toxic lipid alcohols under physiological conditions (Yang et al., 2014; Friedmann Angeli et al., 2014; Wang et al., 2012). Radiation depletes intracellular GSH by suppressing cystine transport and simultaneously inactivates GPX4 through sustained ROS production. The temporal convergence of NCOA4-mediated ferritinophagy and GPX4 dysfunction reveals a synergistic regulatory mechanism: ferritin degradation expands the labile iron pool, whereas GPX4 inactivation abolishes lipid peroxide detoxification, jointly driving an irreversible lipid peroxidation cascade (Zhou et al., 2022; Lai et al., 2025; Ji et al., 2022). In addition, the ferroptosis suppressor protein 1 (FSP1)-coenzyme Q10 and GTP cyclohydrolase 1 (GCH1)-tetrahydrobiopterin (BH_4_) pathways serve as auxiliary antioxidant defense systems (Sun et al., 2022; Bersuker et al., 2019); however, radiation impairs these protective systems, further shifting the cellular redox balance toward ferroptotic cell death (Yan et al., 2020).

Mitochondria and the ER function as central execution platforms for NCOA4-driven ferroptosis under radiation stress. Excessive mitochondrial iron accumulation increases superoxide production, triggering cardiolipin oxidation and mitochondrial permeability transition (Ito et al., 2021; Abe et al., 2022). Concurrently, ER oxidative stress activates the unfolded protein response (UPR), and downstream mediators such as CHAC1 further deplete intracellular GSH, thus weakening cellular antioxidant defenses. Moreover, ER stress promotes mitochondrial Ca^2+^ influx through IP3R at ER-mitochondria contact sites, elevating mitochondrial ROS levels and lowering the mitochondrial permeability transition threshold, which increases cellular susceptibility to ferroptosis (Cam et al., 2024). Radiation amplifies this inter-organelle crosstalk by promoting the formation of mitochondrial-associated membranes, enabling the direct transfer of ferrous ions and ROS between subcellular compartments (Mancias et al., 2014; Zhou et al., 2022; Wenzel et al., 2017; Ito et al., 2021). These coordinated interactions transform localized oxidative insults into a system-wide ferroptotic response by coupling ferritinophagy-mediated iron mobilization with lipid peroxidation execution.

Collectively, these molecular effectors constitute an integrated mechanistic axis that links ferritin autophagy to ferroptosis execution. NCOA4-mediated ferritin degradation initiates intracellular iron mobilization, the ALOX-ACSL4-LPCAT3 pathway amplifies lipid peroxidation progression, and GPX4 inactivation commits cells to ferroptotic death. Rather than operating as a linear cascade, this process functions as a self-reinforcing biochemical circuit, in which iron trafficking, lipid metabolic reprogramming, and antioxidant defense collapse are tightly coordinated both temporally and spatially, integrating redox metabolism with regulated cell death under radiation stress.

## 6 Crosstalk between ferroptosis and other radiation-induced cell death pathways

Radiotherapy initiates multiple regulated cell death programs, including apoptosis, necroptosis, pyroptosis, autophagy-dependent cell death, and ferroptosis (Stockwell, 2022; Galluzzi et al., 2018; Tang et al., 2019). The relative contribution of each pathway is determined by radiation dose, fractionation regimen, tumor type, and the cellular microenvironment (Stockwell, 2022; Tang et al., 2019). Although these cell death modalities possess distinct molecular characteristics, they frequently coexist in irradiated tumors and collectively determine therapeutic efficacy (Stockwell, 2022; Tang et al., 2019).

Rather than acting as completely independent processes, these cell death pathways are interconnected via multiple common upstream signals. Ionizing radiation induces excessive ROS production, DNA damage, mitochondrial dysfunction, and inflammatory responses, all of which simultaneously modulate multiple regulated cell death programs (Stockwell, 2022; Lei et al., 2022; Sun et al., 2023; Tang et al., 2019). For instance, ROS act as central mediators of both apoptosis and ferroptosis, whereas DNA damage activates signaling pathways that regulate apoptosis, ferroptosis, and necroptosis (Lei et al., 2022; Sun et al., 2023; Tang et al., 2019). Similarly, mitochondrial dysfunction and inflammatory signaling are involved in coordinating the activation of multiple forms of radiation-induced cell death (Stockwell, 2022; Lei et al., 2022; Sun et al., 2023). These findings indicate that extensive molecular crosstalk occurs among distinct cell death pathways following irradiation (Stockwell, 2022; Lei et al., 2022; Sun et al., 2023; Tang et al., 2019).

To date, however, the most robust evidence supports the role of NCOA4-mediated ferritinophagy in regulating ferroptosis through the mobilization of ferritin-bound iron and the expansion of the intracellular labile iron pool (Mancias et al., 2014; Wang et al., 2023a). Whether NCOA4 directly interacts with apoptosis, pyroptosis, or necroptosis remains largely unclear (Mancias et al., 2014; Wang et al., 2023a). Although ferritinophagy-mediated iron release may indirectly affect oxidative stress, mitochondrial function, or inflammatory signaling, definitive mechanistic evidence linking NCOA4 to these alternative cell death programs is currently scarce (Mancias et al., 2014; Wang et al., 2023a). Future studies should therefore explore whether NCOA4-mediated ferritinophagy functions as a central coordinator of multiple radiation-induced cell death pathways or acts primarily as a ferroptosis-specific regulatory mechanism (Wang et al., 2023a). At present, available evidence does not support a direct regulatory role of NCOA4 in apoptosis, pyroptosis, or necroptosis that is comparable to its well-characterized function in ferroptosis, highlighting a critical knowledge gap for future research (Mancias et al., 2014; Wang et al., 2023a).

## 7 Regulatory mechanisms limiting iron mobilization and lipid peroxidation during radiotherapy

Although NCOA4-mediated ferritinophagy serves as a key driver of radiation-induced ferroptosis, its activity is tightly controlled by multilayered negative feedback mechanisms that restrict iron mobilization, buffer labile ferrous ions, and inhibit excessive lipid peroxidation. These intrinsic protective checkpoints, including iron sequestration and export pathways, antioxidant repair systems, and adaptive modulation of autophagy, collectively define the ferroptotic threshold and modulate cellular sensitivity to radiation (Mancias et al., 2014; Fang et al., 2021; Lang et al., 2019; Yang et al., 2014; Ji et al., 2022).

At the level of iron homeostasis, a primary counter-regulatory response involves ferritin resynthesis, controlled by the Nrf2-FTH1/FTL axis. After radiation-triggered ferritin degradation, Nrf2 activation promotes the transcription of FTH1 and FTL, recovering ferritin function, buffering free iron, and restricting expansion of the labile iron pool (Zhou et al., 2022; Anandhan et al., 2023; Savic et al., 2024). Meanwhile, the iron exporter SLC40A1 drives cytosolic Fe^2+^ efflux to sustain redox balance, and its upregulation under sublethal radiation stress acts as a key adaptive survival mechanism (Mansuer et al., 2024). Besides, mitochondrial ferritin (FtMt) serves as a compartment-specific safeguard by trapping ferrous iron inside the mitochondrial matrix, which suppresses Fenton chemistry and blocks cardiolipin oxidation. The absence of FtMt causes mitochondrial iron overload, intensified lipid peroxidation, and higher cellular vulnerability to radiation-induced ferroptosis, which confirms its status as a vital spatial checkpoint in iron regulation (Song et al., 2024).

An extra regulatory layer lies in the selective modulation of NCOA4 activity. Post-translational changes greatly affect NCOA4 stability and its interaction with ferritin, thus altering the overall level of ferritinophagy. Existing research shows that ATM-mediated phosphorylation improves NCOA4-ferritin binding and accelerates ferritin breakdown, while SUMOylation modulates NCOA4 proteostability and works as a molecular switch to control ferritin turnover (Wu et al., 2023a; Xue et al., 2025). In addition, mTORC1 limits ferritinophagy indirectly by inhibiting autophagic processes; yet it remains unclear whether mTORC1 can directly disrupt the NCOA4-ferritin complex via phosphorylation, which requires further research (Du et al., 2024; Koshizuka et al., 2025). Under mild oxidative stress, heat shock protein B1 (HSPB1) maintains the stability of ferritin complexes and prevents NCOA4 from recruiting to autophagosomes, which slows down iron release (Zhang et al., 2023b; Mishima et al., 2022). On the contrary, persistent metabolic stress induced by radiation or pharmacological mTORC1 inhibition removes these restrictive effects, turning NCOA4 from a strictly controlled iron-buffering factor into a strong pro-ferroptotic mediator (Mancias et al., 2014; Zhou et al., 2022; Ito et al., 2021). Taken together, these feedback mechanisms allow cells to dynamically adjust ferritinophagy according to the intensity and duration of radiation-triggered oxidative stress.

At the lipid peroxidation checkpoint, the suppression of ferroptosis is mainly achieved through the GPX4-GSH axis and the independent FSP1-CoQ10 system. GPX4 uses GSH as an electron donor to neutralize phospholipid hydroperoxides (PE-OOH), while FSP1 functions on the plasma membrane to regenerate reduced coenzyme Q10, eliminating lipid radicals and stopping peroxidative chain reactions (Yang et al., 2014; Friedmann Angeli et al., 2014; Bersuker et al., 2019; Mishima et al., 2022). Ionizing radiation disrupts both protective pathways by depleting intracellular NADPH and cysteine. Nevertheless, cells that survive acute oxidative damage can restore system Xc^−^ transport function, replenish cysteine reserves, and maintain GSH biosynthesis (Tang et al., 2021; Lang et al., 2019; Jomova et al., 2023). In line with this protective effect, forced upregulation of SLC7A11 or FSP1 markedly reduces radiation-induced ferroptosis, proving that these pathways are core biochemical constraints that determine the resistance threshold of the ferroptosis machinery (Reinheckel and Tholen, 2022; Yan et al., 2020).

Beyond enzymatic antioxidant defenses, membrane lipid remodeling provides an additional structural barrier against ferroptosis. Acyl-CoA synthetase long-chain family member 3 (ACSL3) and stearoyl-CoA desaturase 1 (SCD1) cooperate to regulate monounsaturated fatty acid (MUFA) metabolism. To be specific, ACSL3 preferentially activates MUFAs for incorporation into phospholipids, while SCD1 converts saturated fatty acids into MUFAs. Through the enrichment of MUFA-containing phospholipids, susceptibility to iron-driven peroxidation is reduced, and the replacement of polyunsaturated fatty acid-phospholipid (PUFA-PL) species is enabled within membrane bilayers. Thus, lipid peroxidation and ferroptotic cell death can be limited (Magtanong et al., 2019; Tesfay et al., 2019; Worthmann et al., 2024). In irradiated cancer cells, SCD1 upregulation is associated with ferroptosis resistance and increased radio-tolerance, whereas pharmacological inhibition of SCD1 restores a PUFA-rich membrane composition and resensitizes cells to ferroptosis (Magtanong et al., 2019; Tesfay et al., 2019; Luo et al., 2022).

An additional regulatory layer involves autophagy-lysosome-mediated feedback control. The adaptor protein p62/sequestosome 1 (p62/SQSTM1) regulates the turnover of Kelch-like ECH-associated protein 1 (Keap1)-nuclear factor erythroid 2-related factor 2 (Nrf2), thereby activating transcriptional programmes that promote antioxidant defense and iron sequestration (Lee et al., 2020; Feng et al., 2024). During sustained ferritin autophagy, p62 phosphorylation induces transient Nrf2 activation; however, excessive autophagic flux can ultimately overwhelm this protective response, shifting the cellular balance toward ferroptosis (Irimia-Dominguez et al., 2020; Zhao et al., 2024a; Feng et al., 2024; Ikeda et al., 2023; Li et al., 2022). In parallel, lysosomal stabilization achieved via cathepsin inhibition or treatment with iron chelators including deferoxamine (DFO) and ciclopirox (CPX) restricts ferrous iron leakage and suppresses the propagation of lipid peroxidation (Delanoy et al., 2024; Saimoto et al., 2025; Nag et al., 2021).

**FIGURE 4:**
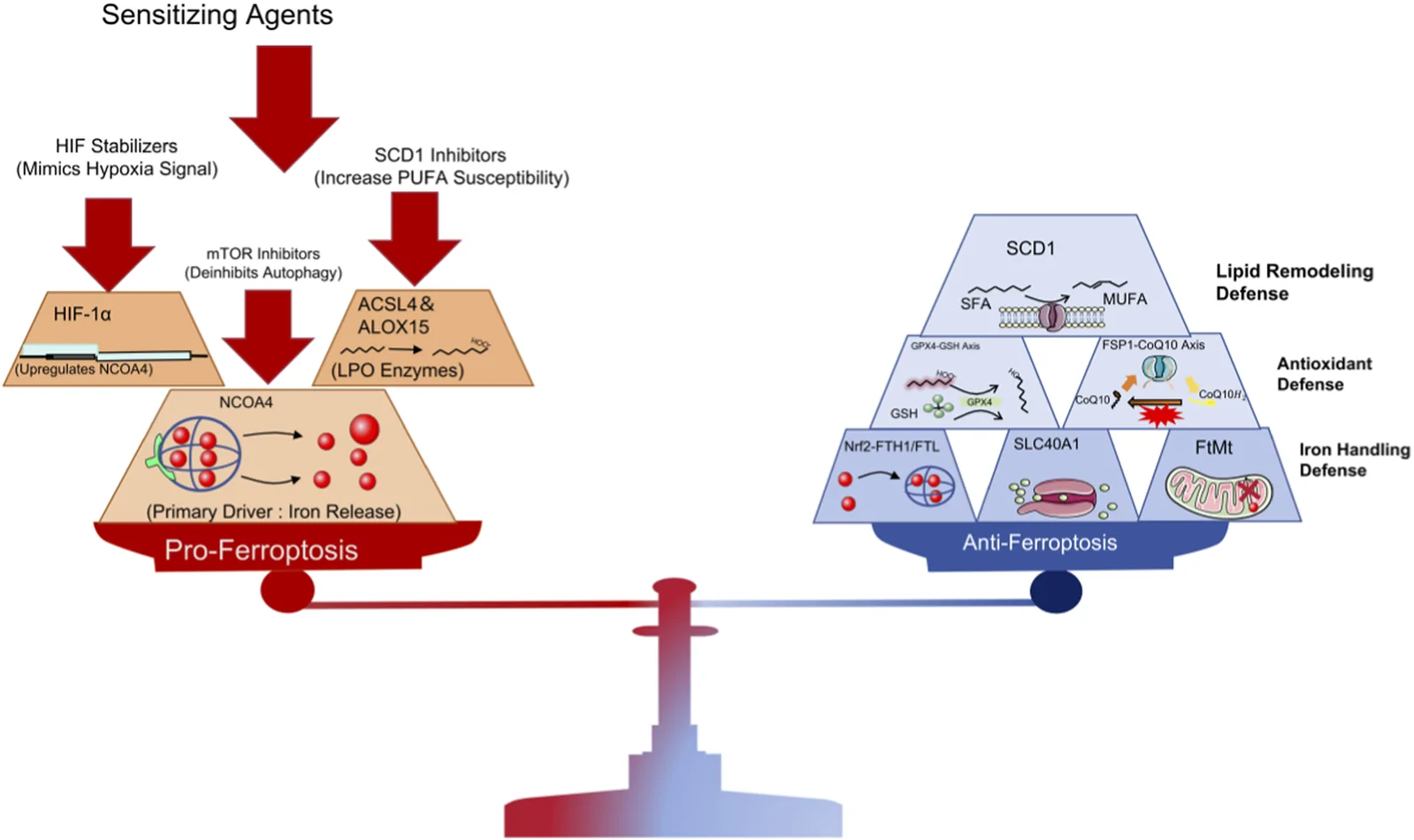
Potential therapeutic strategies for modulating ferroptosis during radiotherapy. Ferroptosis regulation represents a potential approach to improve the therapeutic window of radiotherapy. In tumor cells, strategies that enhance iron mobilization, promote ferroptotic signaling, or inhibit antioxidant defense pathways may increase radiosensitivity. Candidate approaches include modulation of NCOA4-mediated ferritinophagy and targeting ferroptosis defense systems such as GPX4, FSP1, and SLC7A11. Conversely, protection of normal tissues may be achieved by limiting excessive iron-dependent oxidative damage through iron chelation, NRF2 activation, preservation of ferritin-mediated iron storage, and reinforcement of antioxidant capacity. These therapeutic concepts remain largely supported by preclinical studies and require further evaluation regarding tumor selectivity, safety, and clinical feasibility.

In summary, these negative regulatory mechanisms collectively establish an adaptive equilibrium between ferroptosis and anti-ferroptotic defenses. The coordinated activation of these regulatory checkpoints determines whether irradiated cells undergo irreversible ferroptotic death or restore iron and redox homeostasis for survival. Characterizing the context-dependent activation of these protective circuits in tumor tissues relative to normal tissues provides a mechanistic basis for the selective modulation of ferroptosis, thereby enhancing tumor radiosensitization while preserving adjacent healthy tissues (Figure 4).

## 8 Key controversies and knowledge gaps

Despite substantial advances in the characterization of ferritin autophagy-mediated ferroptosis in radiation biology, several critical mechanistic questions remain unresolved. These knowledge gaps underscore the complex regulation of iron dynamics and lipid peroxidation under heterogeneous radiotherapy conditions, posing substantial challenges for the clinical translation of ferroptosis-targeted therapeutic interventions.

Recent studies have elucidated a mechanistic link between the DNA damage response (DDR) and iron metabolism via the modulation of ferritinophagy. In particular, accumulating evidence indicates that the serine/threonine kinase ataxia-telangiectasia mutated (ATM) may directly phosphorylate and regulate the ferritinophagy receptor NCOA4. A recent mechanistic study demonstrated that ATM phosphorylates NCOA4 and strengthens its interaction with ferritin heavy chain (FTH1), thereby facilitating ferritin degradation and elevating intracellular labile iron levels to drive ferroptotic lipid peroxidation (Wu et al., 2023a). Genetic deletion or pharmacological inhibition of ATM markedly suppresses ferritinophagy and confers ferroptosis resistance, confirming that ATM acts as an upstream regulator that bridges DDR signaling to iron-dependent oxidative cell death pathways (Wu et al., 2023a). Consistent with this regulatory model, additional experimental evidence verifies that ATM-dependent phosphorylation enhances NCOA4-ferritin binding and promotes ferritin turnover under conditions of oxidative stress or metabolic injury (Jiang et al., 2024).

However, whether ATM directly phosphorylates NCOA4 in the context of radiation-induced DDR extends beyond a purely biochemical question and instead reflects a broader issue regarding DDR signaling specificity. Within the DDR network, ATM, ATR, and DNA-PKcs constitute the three principal phosphatidylinositol-3-kinase-related kinases (PIKKs) that orchestrate cellular responses to DNA damage (Wang et al., 2023a). While ATM and DNA-PKcs are predominantly activated by DNA DSBs, ATR is typically activated by single-stranded DNA intermediates generated during DNA end resection and replication stress (Wang et al., 2023a; Blackford and Jackson, 2017). As indicated by accumulating evidence, substantial substrate overlap and signaling crosstalk exist among these kinases. Thus, the possibility that NCOA4 phosphorylation may not be exclusively mediated by ATM can be elevated, particularly under complex genotoxic conditions such as ionizing radiation exposure (Wang et al., 2023a; Blackford and Jackson, 2017).

Radiation quality may further modulate the upstream signaling pathways that regulate NCOA4. High-linear energy transfer (LET) radiation, such as carbon ions or α-particles, induces clustered and complex DNA damage that preferentially activates ATM- and DNA-PK-dependent DSB signaling pathways. In contrast, low-LET radiation (e.g., X-rays or γ-rays) more frequently generates dispersed DNA lesions that undergo end resection, leading to robust ATR-dependent checkpoint activation, particularly during S-phase progression (Blackford and Jackson, 2017; Araszkiewicz et al., 2025; Yang et al., 2025; Kuno and Iwai, 2023). These distinctions suggest that different radiation modalities may trigger distinct DDR kinase hierarchies, which could in turn differentially regulate NCOA4-dependent ferritinophagy and ferroptosis. Therefore, clarifying whether ATR or DNA-PKcs can also phosphorylate NCOA4, and determining whether this regulatory pattern varies according to radiation quality or LET, represent key unresolved questions for understanding how the DDR-iron metabolism axis mediates radiation-induced ferroptotic signaling.

An important yet largely unexplored dimension of radiation-induced ferroptosis is the influence of irradiation modality and dose rate, particularly in the context of FLASH radiotherapy. Current mechanistic insights into radiation-related ferroptosis are derived almost exclusively from studies adopting conventional dose-rate irradiation, while the molecular outcomes of ultra-high dose-rate (FLASH) irradiation remain poorly characterized. FLASH irradiation delivers radiation within microseconds to milliseconds and generates unique radiochemical and redox conditions, including transient oxygen depletion and altered radical recombination kinetics (Abiri et al., 2019; Mateo et al., 2019). These distinct physicochemical properties may profoundly modulate the iron-dependent lipid peroxidation pathways that underpin ferroptosis.

From a mechanistic perspective, FLASH irradiation may alter the canonical NCOA4-mediated ferritinophagy pathway, which is currently recognized as a major source of labile iron that drives ferroptotic lipid peroxidation. Rapid oxygen depletion during FLASH exposure may transiently suppress ROS-dependent iron mobilization, thereby restricting ferritin degradation or delaying NCOA4 activation. Alternatively, the ultra-high dose rate may trigger distinct intracellular iron redistribution, such as lysosomal iron sequestration or mitochondrial iron accumulation, processes closely implicated in the modulation of ferroptosis sensitivity under oxidative stress conditions (Stillman et al., 2020; Rizzoll et al., 2025). These considerations raise the possibility that FLASH irradiation may partially bypass NCOA4-dependent ferritinophagy, and instead activate alternative mechanisms of iron homeostasis regulation or redox adaptation.

Notably, direct experimental evidence regarding FLASH-induced ferroptosis and NCOA4 regulatory mechanisms remains extremely limited, constituting a critical knowledge gap within this research field. Given that ferroptosis is recognized as a key mediator of radiation-triggered tumor cell death and normal tissue toxicity, clarifying whether ultra-high dose-rate irradiation remodels iron metabolism, lipid peroxidation dynamics, or ferroptotic thresholds is essential for deciphering the biological effects of FLASH therapy. Future studies combining dose-rate-controlled irradiation systems, high-resolution iron imaging techniques, and ferroptosis-specific molecular biomarkers are warranted to clarify whether FLASH irradiation modulates the DDR-iron metabolism axis and consequently alters the ferroptotic susceptibility of irradiated tissues.

Moreover, the spatiotemporal characteristics of subcellular iron redistribution following different irradiation modalities remain poorly characterized. Most existing mechanistic findings are obtained from conventional fractionated radiotherapy, while high-dose and FLASH irradiation create unique redox microenvironments that may fundamentally reshape ferroptotic signaling cascades (Mancias et al., 2015; Ma et al., 2021; Vozenin et al., 2024). Accumulating evidence demonstrates that ultra-high-dose irradiation triggers transient lysosomal iron retention, which restricts ferrous ion release and inhibits ferroptosis, whereas prolonged irradiation induces persistent mitochondrial iron accumulation and exacerbates lipid peroxidation (Yang et al., 2023; Froidevaux et al., 2023). Whether these opposing biological outcomes stem from altered oxygen depletion kinetics, modified NCOA4 activation thresholds, or dose-dependent regulation of autophagic turnover remains unclear and necessitates systematic exploration.

Tumor heterogeneity further complicates the clarification of the functional roles of NCOA4-mediated ferritinophagy in tumor biology. Distinct tumor types exhibit significant differences in basal iron metabolism, lipid profiles, and antioxidant capability, all of which serve as core determinants of cellular ferroptosis susceptibility. For instance, tumors with upregulated transferrin receptor expression or enhanced mitochondrial iron utilization display aberrant ferritin turnover and iron trafficking, biological processes tightly governed by NCOA4-dependent ferritinophagy (Wang et al., 2023a). Nevertheless, the functional outcomes of this pathway are highly context-specific. In pancreatic ductal adenocarcinoma, NCOA4-mediated ferritinophagy supports tumor progression by sustaining iron bioavailability for mitochondrial iron-sulfur cluster protein synthesis and oxidative metabolism (Santana-Codina et al., 2022). In contrast, in other tumor backgrounds, elevated NCOA4-mediated ferritinophagy facilitates iron release from ferritin and renders tumor cells more vulnerable to ferroptotic lipid peroxidation, highlighting the dual, tumor type-specific functions of this pathway in cancer progression and therapeutic responsiveness (Shen et al., 2024).

In addition to intrinsic metabolic heterogeneity, the TME is increasingly acknowledged as a critical modulator of ferroptosis sensitivity. Immune and stromal components within the TME, including tumor-associated macrophages (TAMs) and cancer-associated fibroblasts (CAFs), can substantially reshape tumor iron metabolism and redox homeostasis via the secretion of cytokines, metabolic intermediates, and extracellular vesicles (Gu et al., 2026). As key participants in systemic and local iron recycling, macrophages can release iron-containing molecules and inflammatory mediators that tune oxidative stress levels and ferroptosis susceptibility in tumor cells. Growing evidence further reveals that extracellular vesicles and exosomes facilitate the intercellular transfer of ferritin and iron-related molecules, thereby remodeling intracellular iron reservoirs and ferroptotic signaling pathways in recipient cells (Huang et al., 2025). These intercellular communication mechanisms raise the possibility that microenvironment-derived signals may indirectly affect NCOA4 stability, ferritin turnover, or ferritin phase separation dynamics, ultimately modulating ferritinophagy activity within tumor cells.

Collectively, these findings demonstrate that the functional role of NCOA4 cannot be fully elucidated without considering both intrinsic tumor metabolic heterogeneity and extrinsic microenvironmental regulation. Future studies should therefore investigate whether microenvironmental cues, including macrophage-derived cytokines, fibroblast-secreted factors, and extracellular vesicle-mediated iron transfer, directly regulate NCOA4 expression, post-translational modifications, or ferritin-binding capability. Such research is essential to clarify how the NCOA4-ferritinophagy axis integrates tumor metabolism with the TME to govern ferroptosis sensitivity and therapeutic responsiveness.

In summary, resolving these unresolved questions is critical for advancing ferritinophagy-driven ferroptosis from a theoretical radiobiological framework toward a clinically viable therapeutic strategy.

## 9 Conclusion

Emerging evidence establishes NCOA4-mediated ferritinophagy as a vital regulatory node that bridges radiation-induced DNA damage responses, intracellular iron homeostasis, and ferroptotic susceptibility. By modulating the magnitude and dynamics of the intracellular labile iron pool, cells initiate transient adaptive antioxidant responses post irradiation or undergo persistent ferrous iron accumulation, which augments lipid peroxidation and triggers ferroptotic cell death (Mancias et al., 2015; Zhou et al., 2022; Anandhan et al., 2023; Wu et al., 2023a; Ma et al., 2021). Recent work further shows that sustained ferritin autophagy depletes endogenous iron-buffering capacity and impairs redox homeostasis, thereby driving irreversible cellular transition toward ferroptosis. This integrated process mechanistically links DNA damage signaling, autophagic flux regulation, and lipid oxidative metabolism to form a coordinated ferroptotic cascade (Mancias et al., 2015; Lei et al., 2020; Xie et al., 2016; Wu et al., 2023a; Ma et al., 2021).

From a translational perspective, the ferroptosis regulatory network offers a solid conceptual basis for the development of next-generation radiotherapeutic regimens. Therapeutic upregulation of ferritin autophagy or blockade of ferroptosis-suppressive pathways may resensitize radio- and drug-resistant tumors, while transient suppression of NCOA4 activity and reinforcement of iron-sequestration mechanisms can shield normal tissues against radiation injury (Zhou et al., 2022; Lang et al., 2019; Bersuker et al., 2019). Such bidirectional therapeutic potential highlights the necessity of context-specific, precise modulation of ferroptosis, rather than generalized adoption of traditional cytotoxic treatment strategies.

**FIGURE 5:**
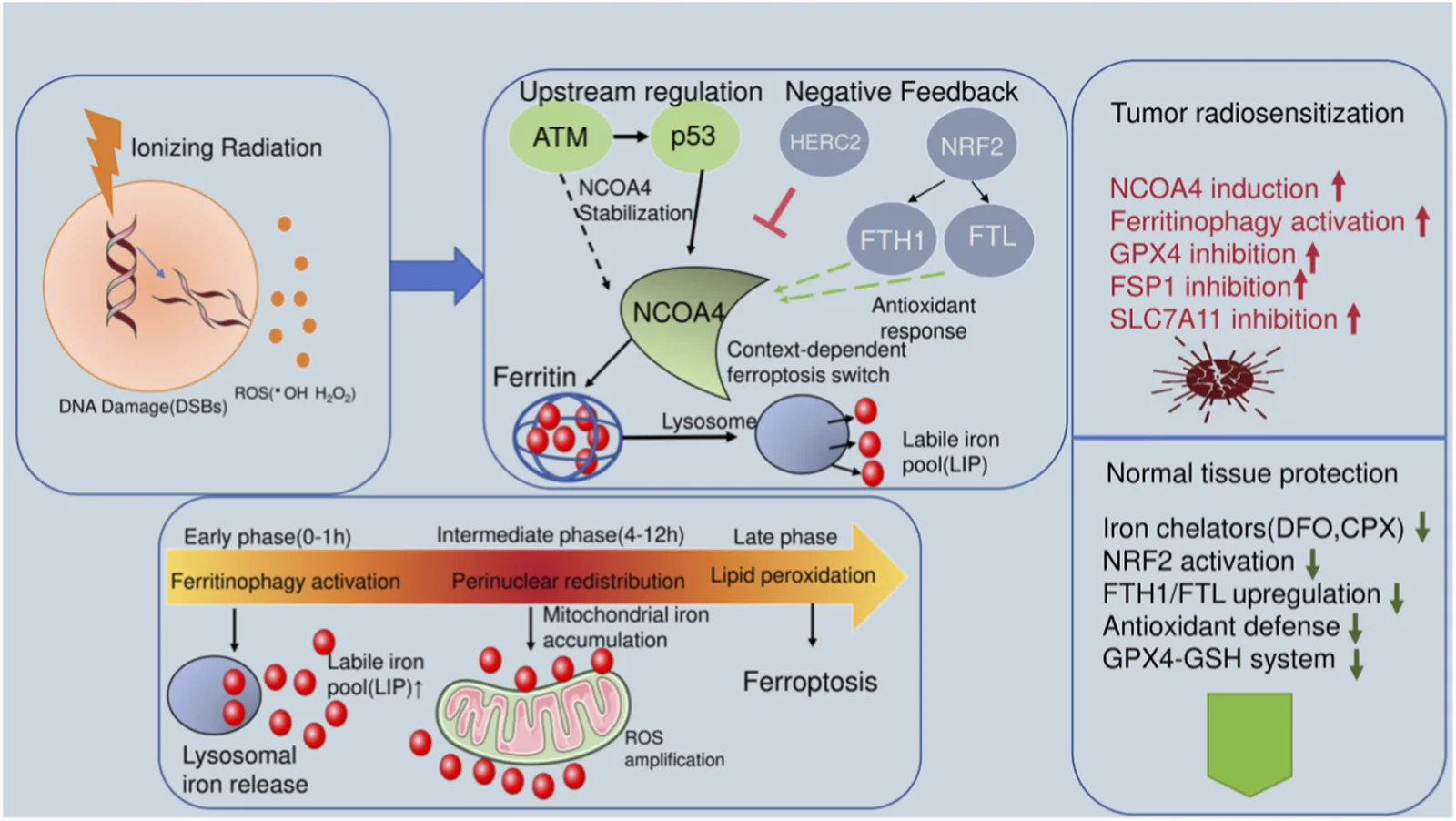
Integrated framework linking radiation stress, iron remodeling, ferroptosis regulation, and therapeutic opportunities. This schematic summarizes the interconnected processes underlying radiation-induced ferroptosis and highlights the regulatory contribution of NCOA4-mediated ferritinophagy within a broader network of iron metabolism, oxidative stress responses, and ferroptosis control. Ionizing radiation initiates DNA damage and ROS generation, which activate multiple signaling pathways involved in ferroptotic regulation. NCOA4-mediated ferritinophagy contributes to ferritin degradation and labile iron pool expansion, while negative regulatory mechanisms, including HERC2-mediated NCOA4 degradation and NRF2-associated antioxidant responses, help maintain iron and redox homeostasis. The proposed spatiotemporal progression involves early iron release, subsequent intracellular iron redistribution, and lipid peroxidation-associated ferroptotic execution. Therapeutically, modulation of ferroptosis may provide opportunities for tumor radiosensitization or normal tissue protection; however, these strategies remain primarily preclinical and require further validation regarding specificity, toxicity, and clinical translation.

Despite these research advances, multiple key questions remain unaddressed. It remains elusive whether NCOA4 activation serves as a universal hallmark of radiation-induced ferroptosis, or whether its functional contribution varies with tumor type, genetic background, metabolic status and irradiation modality. Furthermore, the molecular hierarchy connecting ATM signaling, p53 activation, and NCOA4-mediated ferritinophagy requires further mechanistic validation in a wide range of experimental models. From a translational standpoint, selective targeting of the NCOA4-ferritinophagy axis poses substantial challenges, given the indispensable role of ferritinophagy in maintaining physiological iron homeostasis in normal tissues. Future research should focus on screening predictive biomarkers, establishing tumor-selective delivery approaches, and defining the therapeutic window for ferritinophagy modulation, so as to maximize tumor radiosensitization while mitigating normal tissue toxicity. Resolving these outstanding issues is essential to translate NCOA4-targeted ferroptosis modulation into clinical precision radiotherapy (Figure 5).

## Methods

A comprehensive literature search was conducted using PubMed, Web of Science, and Scopus to identify studies published up to February 2026. Keywords included “NCOA4”, “ferritinophagy”, “ferroptosis”, “radiotherapy”, “ionizing radiation”, “iron metabolism”, “lipid peroxidation”, and “DNA damage response”. Only English-language articles with mechanistic relevance were included, while duplicate records, conference abstracts, and non-peer-reviewed studies were excluded. Relevant studies were qualitatively analysed and integrated using a mechanism-oriented narrative approach, with a focus on the regulatory role of NCOA4-mediated ferritinophagy in linking DNA damage response to iron-dependent lipid peroxidation and ferroptosis. Emphasis was placed on signalling pathways, spatiotemporal iron dynamics, and their implications for radiotherapy.

## Glossary

ACSL3: Acyl-CoA synthetase long-chain family member 3
ACSL4: Acyl-CoA synthetase long-chain family member 4
ADCY10: Adenylate cyclase 10
ALOX15: Arachidonate 15-lipoxygenase
AMPK: AMP-activated protein kinase
ATM: Ataxia-telangiectasia mutated
ATR: Ataxia telangiectasia and Rad3-related
BH4: Tetrahydrobiopterin
CAF: Cancer-associated fibroblast
CHAC1: ChaC glutathione specific gamma-glutamylcyclotransferase 1
CoQ10: Coenzyme Q10
DDR: DNA damage response
DFO: Deferoxamine
DMT1: Divalent metal transporter 1
DNA-PKcs: DNA-dependent protein kinase catalytic subunit
DSB: DNA double-strand break
ER: Endoplasmic reticulum
FLASH: Ultra-high dose rate irradiation
FSP1: Ferroptosis suppressor protein 1
FTL: Ferritin light chain
FTH1: Ferritin heavy chain 1
FtMt: Mitochondrial ferritin
GCH1: GTP cyclohydrolase 1
GPX4: Glutathione peroxidase 4
GSH: Glutathione
HERC2: HECT and RLD domain containing E3 ubiquitin protein ligase 2
HIF-1α: Hypoxia-inducible factor 1 alpha
IP3R: Inositol 1,4,5-trisphosphate receptor
IR: Ionizing radiation
LET: Linear energy transfer
LIP: Labile iron pool
LMP: Lysosomal membrane permeabilization
LPCAT3: Lysophosphatidylcholine acyltransferase 3
MAMs: Mitochondria-associated membranes
mTORC1: Mechanistic target of rapamycin complex 1
MUFA: Monounsaturated fatty acid
NCOA4: Nuclear receptor coactivator 4
Nrf2: Nuclear factor erythroid 2–related factor 2
PCBP1: Poly(rC)-binding protein 1
PCBP2: Poly(rC)-binding protein 2
PIKKs: Phosphatidylinositol 3-kinase-related kinases
PUFA: Polyunsaturated fatty acid
PUFA-PE: Polyunsaturated fatty acid–phosphatidylethanolamine
ROS: Reactive oxygen species
SCD1: Stearoyl-CoA desaturase 1
SLC7A11: Solute carrier family 7 member 11
SLC40A1: Solute carrier family 40 member 1
SUMO: Small ubiquitin-like modifier
**System Xc** ^-^: Cystine/glutamate antiporter system
TAM: Tumor-associated macrophage
TFEB: Transcription factor EB
TFRC: Transferrin receptor
TME: Tumor microenvironment
ULK1: Unc-51 like autophagy activating kinase 1
UPR: Unfolded protein response
VDAC1: Voltage-dependent anion channel 1
YAP: Yes-associated protein
